# Supplementary material for: Bayesian tip dating reveals heterogeneous morphological clocks in Mesozoic birds
Source: R Soc Open Sci. 2019 Jul 24;6(7):182062. doi: 10.1098/rsos.182062 (PMC6689603; doi:10.1098/rsos.182062)
Supplement: Character Description [file rsos182062supp1.pdf]

Character description

1. Premaxillae in adults: unfused (0); fused only rostrally (1); completely fused (2). (ORDERED)
2. Maxillary process of the premaxilla: restricted to its rostral portion (0); subequal or longer than the facial contribution of the maxilla (1).
3. Frontal process of the premaxilla: short (0); relatively long, approaching the rostral border of the antorbital fenestra (1); very long, extending caudally near the level of lacrimals (2). (ORDERED)
4. Premaxillary teeth: present throughout (0); present but rostral tip edentulous (1); present but restricted to rostral portion (2); absent (3).
5. Caudal margin of naris: far rostral than the rostral border of the antorbital fossa (0); nearly reaching or overlapping the rostral border of the antorbital fossa (1).
6. Naris longitudinal axis: considerably shorter than the long axis of the antorbital fossa (0); subequal or longer (1).
7. Maxillary teeth: present (0); absent (1).
8. Dorsal (ascending) ramus of the maxilla: present with two fenestrae (the promaxillary and maxillary fenestra) (0); present with one fenestra (1); unfenestrated (2); ramus absent (3). (ORDERED)
9. Caudal margin of choana: located rostrally, not overlapping the region of the orbit (0); displaced caudally, at the same level or overlapping the rostral margin of the orbit (1).
10. Rostral margin of the jugal: away from the caudal margin of the naris (0); or very close to (leveled with) the caudal margin of the naris (1).
11. Contact between palatine and maxilla/premaxilla: palatine contact maxilla only (0); contacts premaxilla and maxilla (1).
12. Vomer and pterygoid articulation: present, well developed (0); reduced, narrow process of pterygoid passes dorsally over palatine to contact vomer (1); absent, pterygoid and vomer do not contact (2).
13. Jugal process of palatine: present (0); absent (1).
14. Contact between palatine and pterygoid: long, craniocaudally overlapping contact (0); short, primarily dorsoventral contact (1).
15. Contact between vomer and premaxilla: present (0); absent (1).
16. Ectopterygoid: present (0); absent (1).
17. Postorbital: present (0); absent (1).
18. Contact between postorbital and jugal: present (0); absent (1).
19. Quadratojugal: sutured to the quadrate (0); joined through a ligamentary articulation (1).
20. Lateral, round cotyla on the mandibular process of the quadrate (quadratojugal articulation): absent (0); present (1).
21. Contact between the quadratojugal and squamosal: present (0); absent (1).

22. Squamosal incorporated into the braincase, forming a zygomatic process: absent (0); present (1).
23. Squamosal, ventral or "zygomatic" process: variably elongate, dorsally enclosing otic process of the quadrate and extending cranioventrally along shaft of this bone, dorsal head of quadrate not visible in lateral view (0); short, head of quadrate exposed in lateral view (1).
24. Frontal/parietal suture in adults: open (0); fused (1).
25. Quadrate orbital process (pterygoid ramus): broad (0); sharp and pointed (1).
26. Quadrate pneumaticity: absent (0); present (1).
27. Quadrate: articulating only with the squamosal (0); articulating with both prootic and squamosal (1).
28. Otic articulation of the quadrate: articulates with a single facet (squamosal) (0); articulates with two distinct facets (prootic and squamostal) (1); articulates with two distinct facets and quadrate differentiated into two heads (2). (ORDERED)
29. Quadrate distal end: with two transversely aligned condyles (0); with a triangular, condylar pattern, usually composed of three distinct condyles (1).
30. Basipterygoid processes: long (0); short (articulation with pterygoid subequal to, or longer than, amount projected from the basisphenoid rostrum) (1).
31. Pterygoid, articular surface for basipterygoid process: concave "socket", or short groove enclosed by dorsal and ventral flanges (0); flat to convex (1); flat to convex facet, stalked, variably projected (2). (ORDERED)
32. Eustachian tubes: paired, lateral, and well-separated from each other (0); paired, close to each other and to cranial midline or forming a single cranial opening (1).
33. Osseous interorbital septum (mesethmoid): absent (0); present (1).
34. Dentary teeth: present (0); absent (1).
35. Dentary tooth implantation: teeth in individual sockets (0); teeth in a communal groove (1).
36. Symphysial portion of dentaries: unfused (0); fused (1).
37. Deeply notched rostral end of the mandibular symphysis: absent (0); present (1).
38. Mandibular symphysis, symphyseal foramina: absent (0); single (1); paired (2).
39. Mandibular symphysis, symphyseal foramen/foramina: opening on caudal edge of symphysis (0); opening on dorsal surface of symphysis (1).
40. Small ossification present at the rostral tip of the mandibular symphysis (intersymphysial ossification): absent (0); present (1).
41. Caudal margin of dentary strongly forked: unforked, or with a weakly developed dorsal ramus (0); strongly forked with the dorsal and ventral rami approximately equal in caudal extent (1).
42. Mandibular ramus sigmoidal such that the rostral tip is dorsally convex and the caudal end is dorsally concave: absent (0); present (1).
43. Cranial extent of splenial: stops well caudal to mandibular symphysis (0); extending to mandibular symphysis, though non-contacting (1); extending to proximal tip of mandible, contacting on midline (2). (ORDERED)
44. Meckel's groove (medial side of mandible): not completely covered by splenial, deep and conspicuous medially (0); covered by splenial, not exposed medially (1).

45. Rostral mandibular fenestra: absent (0); present (1).
46. Caudal mandibular fenestra: present (0); absent (1).
47. Articular pneumaticity: absent (0); present (1).
48. Teeth: serrated crowns (0); unserrated crowns (1).
49. Atlantal hemiarches in adults: unfused (0); fused, forming a single arch (1).
50. One or more pneumatic foramina piercing the centra of mid-cranial cervicals, caudal to the level of the parapophysis-diapophysis: present (0); absent (1).
51. Cervical vertebrae: variably dorsoventrally compressed, amphicoelous ("biconcave": flat to concave articular surfaces) (0); cranial surface heterocoelous (i.e., mediolaterally concave, dorsoventrally convex), caudal surface flat or slightly concave (1); heterocoelous cranial (i.e., mediolaterally concave, dorsoventrally convex) and caudal (i.e., mediolaterally convex, dorsoventrally concave) surfaces (2). (ORDERED)
52. Prominent carotid processes in the intermediate cervicals: absent (0); present (1).
53. Postaxial cervical epipophyses: prominent, projecting further back from the postzygapophysis (0); weak, not projecting further back from the postzygapophysis, or absent (1).
54. Keel-like ventral surface of cervical centra: absent (0); present (1).
55. Prominent (50% or more the height of the centrum's cranial articular surface) ventral processes of the cervicothoracic vertebrae: absent (0); present (1).
56. Thoracic vertebral count: 13-14 (0); 11-12 (1); fewer than 11 (2). (ORDERED)
57. Thoracic vertebrae: at least part of series with subround, central articular surfaces (e.g., amphicoelous/opisthocoelous) that lack the dorsoventral compression seen in heterocoelous vertebrae (0); series completely heterocoelous (1).
58. Caudal thoracic vertebrae, centra, length and midpoint width: approximately equal in length and midpoint width (0); length markedly greater than midpoint width (1).
59. Wide vertebral foramen in the mid-caudal thoracic vertebrae, vertebral foramen/articular cranial surface ratio (vertical diameter) larger than 0.40: absent (0); present (1).
60. Hyposphene-hypantrum accessory intervertebral articulations in the thoracic vertebrae: present (0); absent (1).
61. Lateral side of the thoracic centra: weakly or not excavated (0); deeply excavated by a groove (1); excavated by a broad fossa (2).
62. Cranial thoracic vertebrae, parapophyses: located in the cranial part of the centra of the thoracic vertebrae (0); located in the central part of the centra of the thoracic vertebrae (1).
63. Notarium: absent (0); present (1).
64. Sacral vertebrae, number ankylosed (synsacrum): less than 7 (0); 7 (1); 8 (2); 9 (3); 10 (4); 11 or more (5); 15 or more (6). (ORDERED)
65. Synsacrum, procoelous articulation with last thoracic centrum (deeply concave facet of synsacrum receives convex articulation of last thoracic centrum): absent (0); present (1).

66. Cranial vertebral articulation of first sacral vertebra: approximately equal in height and width (0); wider than high (1).
67. Series of short sacral vertebrae with dorsally directed parapophyses just cranial to the acetabulum: absent (0); present, three such vertebrae (1); present, four such vertebrae (2). (ORDERED)
68. Convex caudal articular surface of the synsacrum: absent (0); present (1).
69. Degree of fusion of distal caudal vertebrae: fusion absent (0); few vertebrae partially ankylosed (intervening elements are well-discernable) (1); vertebrae completely fused into a pygostyle (2). (ORDERED)
70. Free caudal vertebral count: more than 35 (0); 35-26 (1); 25 - 20 (2); 19-9 (3); 8 or less (4). (ORDERED)
71. Procoelous caudals: absent (0); present (1).
72. Distal caudal vertebra prezygapophyses: elongate, exceeding the length of the centrum by more than 25% (0); shorter (1); absent (2). (ORDERED)
73. Free caudals, length of transverse processes: approximately equal to, or greater than, centrum width (0); significantly shorter than centrum width (1).
74. Proximal haemal arches: elongate, at least 3 times longer than wider (0); shorter (1); absent (2). (ORDERED)
75. Pygostyle: longer than or equal to the combined length of the free caudals (0); shorter (1).
76. Cranial end of pygostyle dorsally forked: absent (0); present (1).
77. Cranial end of pygostyle with a pair of laminar, ventrally projected processes: absent (0); present (1).
78. Distal constriction of pygostyle: absent (0); present (1).
79. Ossified uncinate processes in adults: absent (0); present and free (1); present and fused (2).
80. Uncinate process, orientation: perpendicular to rib (0); angled dorsally defining an acute angle with the rib (1).
81. Gastralia: present (0); absent (1).
82. Coracoid shape: rectangular to trapezoidal in profile (0); strut-like (1).
83. Coracoid and scapula articulation: Scapula and coracoid articulation: (0) pit-shaped scapular cotyla developed on the coracoid, and coracoidal tubercle developed on the scapula (“ball and socket” articulation); (1) scapular articular surface of coracoid convex; (2) flat.
84. Scapula: articulated at the shoulder (proximal) end of the coracoid (0); well below it (1).
85. Coracoid, humeral articular (glenoid) facet: dorsal to acrocoracoid process/“biceps tubercle” (0); ventral to acrocoracoid process (1).
86. Humeral articular facets of the coracoid and the scapula: placed in the same plane (0); forming a sharp angle (1).
87. Coracoid, acrocoracoid: straight (0); hooked medially (1).
88. Laterally compressed shoulder end of coracoid, with nearly aligned acrocoracoid process, humeral articular surface, and scapular facet, in dorsal view: absent (0); present (1).

89. Procoracoid process on coracoid: absent (0); present (1).
90. Lateral margin of coracoid: concave (0); nearly concave to straight for most part and the convex portion is restricted at sternal end, which measures less than half the width of sternal end (1); strongly convex, and the convex portion measuring more than half the sternal end (2).
91. Broad, deep fossa on the dorsal surface of the coracoid (dorsal coracoidal fossa): absent (0); present (1).
92. Supracoracoidal nerve foramen of coracoid: centrally located (0); displaced toward (often as an incisure) the medial margin of the coracoid (1); displaced so that it no longer passes through the coracoid (absent) (2). (ORDERED).
93. Coracoid, medial surface, strongly depressed elongate furrow at the level of the passage of n. supracoracoideus: absent (0); present (1).
94. Supracoracoid nerve foramen, location relative to dorsal coracoidal fossa: above fossa (0); inside fossa (1).
95. Coracoid, sternolateral corner: unexpanded (0); expanded (1); well-developed squared-off lateral process (sternocoracoidal process) (2); present and with a distinct omal projection (hooked) (3).
96. Scapular shaft: straight, both dorsal and ventral margins straight (0); straight shaft with convex dorsal margin and straight ventral margin (1); the scapular shaft sagittally curved (2).
97. Scapula, length: shorter than humerus (0); as long as or longer than humerus (1).
98. Scapular acromion process: in lateral or costal view, strongly projecting craniodorsally, forming a large angle with the proximal shaft of the scapular (0); nearly parallel to the shaft of the scapular (1).
99. Scapula, acromion process: projected cranially surpassing the articular surface for coracoid (0); projected less cranially than the articular surface for coracoid (1).
100. Scapula, acromion process, in costal or lateral aspect: straight and tapered toward cranial end (0); barely tapered with a blunt end (1); laterally hooked tip (2).
101. Proximal end of scapula, pit between acromion and humeral articular facet (scapular fossa): absent (0); present (1).
102. Costal surface of scapular blade with prominent longitudinal furrow: absent (0); present (1).
103. Scapular caudal end: blunt (may or may not be expanded) (0); sharply tapered (1).
104. Furcular, shape: boomerang-shaped (0); V to Y-shaped (1); U-shaped (2).
105. Furcula interclavicular angle: approximately 90° (0); less than 70° (1). The interclavicular angle is measured as the angle formed between three points, one at the omal end of each rami and the apex located at the clavicular symphysis.
106. Dorsal and ventral margins of the furcula: subequal in width (0); ventral margin distinctly wider than the dorsal margin so that the furcular ramus appears concave laterally (1).
107. Hypocleidium: absent (0); present as a tubercle or short process (1); present as an elongate process approximately 30% rami length (2); hypertrophied, exceeding 50% rami length (3). (ORDERED)

108. Sternum: unossified (0); partially ossified, coracoidal facets cartilaginous (1); fully ossified (2).
109. Ossified sternum: two flat plates (0); single flat element (1); single element, with slightly raised midline ridge (2); single element, with projected carina (3).
110. Sternal carina: near to, or projecting rostrally from, the cranial border of the sternum (0); not reaching the cranial border of the sternum (1).
111. Sternum, caudal margin, number of paired caudal trabecula: none (0); one (1); two (2).
112. Sternum, outermost trabecula, shape: tips terminate cranial to caudal end of sternum (0); tips terminate at or approaching caudal end of sternum (1); tips extend caudally past the termination of the sternal midline (2).
113. Prominent distal expansion in the outermost trabecula of the sternum: absent (0); present, simple bulb-like (1); fan-shaped expansion (2); triangular expansion with an acute medial angle (3); branched (4).
114. Rostral margin of the sternum broad and rounded: absent (0); present (1).
115. Sternum, coracoidal sulci spacing on cranial edge: widely separated mediolaterally (0); adjacent (1); crossed on midline (2).
116. Costal facets of the sternum: absent (0); present (1).
117. Sternal costal processes: three (0); four (1); five (2); six (3); seven (4); eight (5). (ORDERED)
118. Sternal midline, caudal end: blunt W-shape (0); V-shape (1); elongate straight projection (xiphoid process) (2); xiphoid process slightly flared mediolaterally (3); xiphoid process distal end strongly flared with prominent medial and lateral projections (4); rounded (5).
119. Sternum, caudal half, paired enclosed fenestra: absent (0); present (1).
120. Sternum, dorsal surface, pneumatic foramen (or foramina): absent (0); present (1).
121. Proximal and distal humeral ends: twisted (0); expanded nearly in the same plane (1).
122. Humeral head: concave cranially and convex caudally (0); globe shaped, craniocaudally convex (1).
123. Proximal margin of the humeral head concave in its central portion, rising ventrally and dorsally: absent (0); present (1).
124. Humerus, proximocranial surface, well-developed circular fossa on midline: absent (0); present (1).
125. Humerus with distinct transverse ligamental groove: absent (0); present (1).
126. Humerus, ventral tubercle projected caudally, separated from humeral head by deep capital incision: absent (0); present (1).
127. Pneumatic fossa in the caudoventral corner of the proximal end of the humerus: absent or rudimentary (0); well developed (1).
128. Humerus, deltopectoral crest: projected dorsally (the plane of the crest is coplanar to the cranial surface of the humerus) (0); projected cranially (1).
129. Humerus, deltopectoral crest: less than shaft width (0); approximately same width (1); prominent and subquadrangular (i.e., subequal length and width) (2).

130. Humerus, deltopectoral crest, perforated by a large fenestra: absent (0); present (1).
131. Humerus, bicipital crest: little or no cranial projection (0); developed as a cranial projection relative to shaft surface in ventral view (1); hypertrophied, rounded tumescence (2).
132. Humerus, distal end of bicipital crest, pit-shaped fossa for muscular attachment: absent (0); craniodistal on bicipital crest (1); directly ventrodistal at tip of bicipital crest (2); caudodistal, variably developed as a fossa (3).
133. Distal end of the humerus very compressed craniocaudally: absent (0); present (1).
134. Humerus, demarcation of muscle origins (e.g., m. extensor metacarpi radialis in Aves) on the dorsal edge of the distal humerus: no indication (0); a pit or a tubercle (1); a variably projected scar-bearing tubercle (dorsal supracondylar process) (2).
135. Well-developed brachial depression on the cranial face of the distal end of the humerus: absent (0); present (1).
136. Well-developed olecranon fossa on the caudal face of the distal end of the humerus: absent (0); present (1).
137. Humerus, distal end, caudal surface, groove for passage of m. scapulotriceps: absent (0); present (1).
138. Humerus, m. humerotricipitalis groove: absent (0); present as a well-developed ventral depression contiguous with the olecranon fossa (1).
139. Humerus, distal margin: approximately perpendicular to long axis of humeral shaft (0); ventrodistal margin projected significantly distal to dorsodistal margin, distal margin angling strongly ventrally (sometimes described as a well-projected flexor process) (1).
140. Humeral distal condyles: mainly located on distal aspect (0); on cranial aspect (1).
141. Humerus, long axis of dorsal condyle: at low angle to humeral axis, proximodistally oriented (0); at high angle to humeral axis, almost transversely oriented (1).
142. Humerus, distal condyles: subround, bulbous (0); weakly defined, “straplike” (1).
143. Humerus, ventral condyle: length of long axis of condyle less than the same measure of the dorsal condyle (0); same or greater (1).
144. Ulna: shorter than humerus (0); nearly equivalent to or longer than humerus (1).
145. Ulnar shaft, radial-shaft/ulnar-shaft ratio: larger than 0.70 (0); smaller than 0.70 (1).
146. Ulna, cotylae: dorsoventrally adjacent (0); widely separated by a deep groove (1).
147. Ulna, dorsal cotyla strongly convex: absent (0); present (1).
148. Ulna, bicipital scar: absent (0); developed as a slightly raised scar (1); developed as a conspicuous tubercle (2).

149. Proximal end of the ulna with a well-defined area for the insertion of m. brachialis anticus: absent (0); present (1).
150. Semilunate ridge on the dorsal condyle of the ulna: absent (0); present (1).
151. Shaft of radius with a long longitudinal groove on its ventrocaudal surface: absent (0); present (1).
152. Ulnare: heart-shaped with little differentiation into short rami (0); U-shaped to V-shaped, well-developed rami (1).
153. Ulnare, ventral ramus: shorter than dorsal ramus (crus brevis) (0); same length as dorsal ramus (1); longer than dorsal ramus (2).
154. Semilunate carpal and proximal ends of metacarpals in adults: unfused (0); semilunate fused to the alular (I) metacarpal (1); semilunate fused to the major (II) and minor (III) metacarpals (2); fusion of semilunate and all metacarpals (3). Any specimen that is inferred to be a juvenile should be scored as a "?" in order to account for the possibility of ontogenetic change.
155. Semilunate carpal, position relative to the alular metacarpal (I): over entire proximal surface (0); over less than one-half proximal surface or no contact present (1).
156. Carpometacarpus, proximal ventral surface: flat (0); raised ventral projection contiguous with minor metacarpal (1); pisiform process forming a distinct peg-like projection (2).
157. Carpometacarpus, ventral surface, supratrochlear fossa deeply excavating proximal surface of pisiform process: absent (0); present (1).
158. Round-shaped alular metacarpal (I): absent (0); present (1).
159. Alular metacarpal (I), extensor process: absent, no cranioproximally projected muscular process (0); present, tip of extensor process just surpassed the distal articular facet for phalanx 1 in cranial extent (1); tip of extensor process conspicuously surpasses articular facet by approximately half the width of facet, producing a pronounced knob (2); tip of extensor process conspicuously surpasses articular facet by approximately the width of facet, producing a pronounced knob (3). (ORDERED)
160. Alular metacarpal (I), distal articulation with phalanx I: ginglymoid (0); shelf (1); ball-like (2).
161. Metacarpal III, craniocaudal diameter as a percentage of same dimension of metacarpal II: approximately equal or greater than 50% (0); less than 50% (1).
162. Proximal extension of metacarpal III: level with metacarpal II (0); ending distal to proximal surface of metacarpal II (1).
163. Intermetacarpal process or tubercle on metacarpal II: absent (0); present as scar (1); present as tubercle or flange (2).
164. Intermetacarpal space: absent or very narrow (0); at least as wide as the maximum width of minor metacarpal (III) shaft (1).
165. Intermetacarpal space: reaches proximally as far as the distal end of metacarpal I (0); terminates distal to end of metacarpal I (1).
166. Distal end of metacarpals: unfused (0); partially or completely fused (1).

167. Minor metacarpal (III) projecting distally more than the major metacarpal (II): absent (0); present (1).
168. Alular digit (I), phalanx 1, distal extension relative to the major metacarpal (II): beyond the distal end of major metacarpal (0); approximately equal in distal extension (1); shorter than the distal end but beyond half of the major metacarpal (2); terminating less than half of the major metacarpal (3). (ORDERED)
169. Proximal phalanx of major digit (II): of normal shape (0); flat and craniocaudally expanded (1).
170. Major digit (II), phalanx 1, "internal index process" (Stegmann, 1978) on caudodistal edge: absent (0); present (1).
171. Second phalanx of major digit (II): longer than proximal phalanx (0); shorter than or equivalent to proximal phalanx (1).
172. Ungual phalanx of major digit (II): present (0); absent (1).
173. Ungual phalanx of major digit (II): larger or subequal to other manual unguals (0); smaller than the alular ungual but larger than that of the minor (III) digit, and the ungual of the minor digit may or may not present (1); smaller than the unguals of the alular and minor digits (2).
174. Proximal phalanx of the minor digit (III) much shorter than the remaining non-ungual phalanges of this digit: absent (0); present (1).
175. Ungual phalanx of minor digit (III): present (0); absent (1).
176. Length of manus (semilunate carpal + major metacarpal and digit) relative to humerus: longer (0); subequal (1); shorter (2). (ORDERED)
177. Intermembral index = (length of humerus + ulna)/(length of femur + tibiotarsus): less than 0.7, flightless (0); between 0.7 and 0.9 (1); between 0.9 and 1.1 (2); greater than 1.1 (3).
178. Pelvic elements in adults, at the level of the acetabulum: unfused or partial fusion (0); completely fused (1).
179. Ilium/ischium, distal co-ossification to completely enclose the ilioischiadic fenestra: absent (0); present (1).
180. Preacetabular process of ilium twice as long as postacetabular process: absent (0); present (1).
181. Preacetabular ilium: approach on midline, open, or cartilaginous connection (0); co-ossified, dorsal closure of "iliosynsacral canals" (1).
182. Ilium, m. cuppedicus fossa as broad, mediolaterally oriented surface directly cranioventral to acetabulum: present (0); surface absent, insertion variably marked by a small entirely lateral fossa cranial to acetabulum (1).
183. Preacetabular pectineal process: absent (0); present as a small flange (1); present as a well-projected flange (2). (ORDERED)
184. Small acetabulum, acetabulum/ilium length ratio equal to or smaller than 0.11: absent (0); present (1).
185. Prominent antitrochanter: caudally directed (0); caudodorsally directed (1).
186. Postacetabular process shallow, less than 50% of the depth of the preacetabular wing at the acetabulum: absent (0); present (1).
187. Iliac brevis fossa: present (0); absent (1).

188. Ischium: two-thirds or less the length of the pubis (0); more than two-thirds the length of the pubis (1).
189. Obturator process of ischium: prominent (0); reduced or absent (1).
190. Ischium, caudal demarcation of the obturator foramen: absent (0); present, developed as a small flange or raised scar contacting/fused with pubis and demarcating the obturator foramen distally (1).
191. Ischium with a proximodorsal (or proximocaudal) process: absent (0); present (1).
192. Ischiadic terminal processes forming a symphysis: present (0); absent (1).
193. Orientation of proximal portion of pubis: cranially to subvertically oriented (0); retroverted, separated from the main synsacral axis by an angle ranging between 65º and 45º (1); more or less parallel to the ilium and ischium (2). (ORDERED)
194. Pubic pedicel: cranioventrally projected (0); ventrally or caudoventrally projected (1).
195. Pubic pedicel of ilium very compressed laterally and hook-like: absent (0), present (1).
196. Pubic shaft laterally compressed throughout its length: absent (0); present (1).
197. Pubic apron: present (0); absent (absence of symphysis) (1).
198. Pubic foot: flaring into simple round shape (0); triangular shape with a pointed caudal tip and caudoventrally directed with respect to the distal pubic shaft (1); the caudal tip recurved caudodorsally with respect to the distal pubic shaft (2); absent (3).
199. Femur with distinct fossa for the capital ligament: absent (0); present (1).
200. Femoral neck: present (0); absent (1).
201. Femoral anterior trochanter: separated from the greater trochanter (0); fused to it, forming a trochanteric crest with a laterally curved edge (1); fused to it, forming a trochanteric crest with a flattened edge (2).
202. Femoral trochanteric crest: projects proximally beyond femoral head (0); equal in proximal projection (1); does not project beyond femoral head (2).
203. Femoral posterior trochanter: present, developed as a slightly projected tubercle or flange (0); hypertrophied, “shelf-like” conformation (1); absent (2).
204. Femur with prominent patellar groove: absent (0); present as a continuous extension onto the distal shaft (1); present and separated from the shaft by a slight ridge, giving it a pocketed appearance (2).
205. Femur: ectocondylar tubercle and lateral condyle separated by deep notch (0); ectocondylar tubercle and lateral condyle contiguous but without developing a tibiofibular crest (1); tibiofibular crest present, defining laterally a fibular trochlea (2). (ORDERED)
206. Caudal projection of the lateral border of the distal end of the femur, proximal and contiguous to the ectocondylar tubercle/tibiofibular crest: absent (0); present (1).
207. Femoral popliteal fossa distally bounded by a complete transverse ridge: absent (0); present (1).
208. Fossa for the femoral origin of m. tibialis cranialis: absent (0); present (1).

209. Tibia, calcaneum, and astragalus: unfused or poorly co-ossified (sutures still visible) (0); complete fusion of tibia, calcaneum, and astragalus (1).
210. Round proximal articular surface of tibiotarsus: absent (0); present (1).
211. Tibiotarsus, proximal articular surface: flat (0); angled so that the medial margin is elevated with respect to the lateral margin (1).
212. Tibiotarsus, cnemial crests: absent (0); present, one (1); present, two (2).
213. Tibia, caudal extension of articular surface for distal tarsals/tarsometatarsus: absent, articular restricted to distalmost edge of caudal surface (0); well-developed caudal extension, sulcus cartilaginis tibialis of Aves, distinct surface extending up the caudal surface of the tibiotarsus (1); with well-developed, caudally projecting medial and lateral crests (2). (ORDERED)
214. Extensor canal on tibiotarsus: absent (0); present as an emarginate groove (1); groove bridged by an ossified supratendinal bridge (2). (ORDERED)
215. Tibia/tarsal-formed condyles: medial condyle projecting farther cranially than lateral condyle (0); equal in cranial projection (1).
216. Tibia/tarsal-formed condyles, mediolateral widths: medial condyle wider (0); approximately equal (1); lateral condyle wider (2). (ORDERED).
217. Tibia/tarsal-formed condyles: gradual sloping of condyles towards midline of tibiotarsus (0); no tapering of either condyle (1).
218. Proximal end of the fibula: prominently excavated by a medial fossa (0); nearly flat (1).
219. Fibula, tubercle for m. iliofibularis: craniolaterally directed (0); laterally directed (1); caudolaterally or caudally directed (2). (ORDERED)
220. Fibula, distal end reaching the proximal tarsals: present (0); absent (1).
221. Distal tarsals in adults: free (0); completely fused to the metatarsals (1). Any specimen that is inferred to be a juvenile should be scored as a "?" in order to account for the possibility of ontogenetic change.
222. Metatarsals II-IV, intermetatarsal fusion: absent or minimal co-ossification (0); partial fusion, sutural contacts easily discernible (1); completely or nearly completely fused, sutural contacts absent or poorly demarcated (2). (ORDERED)
223. Proximal end of metatarsus: plane of articular surface perpendicular to longitudinal axis of metatarsus (0); strongly inclined dorsally (1).
224. Metatarsal V: present (0); absent (1).
225. Proximal end of metatarsal III: in the same plane as metatarsals II and IV (0); plantarly displaced with respect to metatarsals II and IV (1).
226. Tarsometatarsal proximal vascular foramen/foramina: absent (0); one between metatarsals III and IV (1); two (2).
227. Metatarsals, relative mediolateral width: metatarsal IV approximately the same width as metatarsals II and III (0); metatarsal IV narrower than metatarsals II and III (1); metatarsal IV greater in width than either metatarsal II or III (2).
228. Well-developed tarsometatarsal intercotylar eminence: absent (0); present, low and rounded (1); present, high and peaked (2).
229. Tarsometatarsus, projected surface and/or grooves on proximocaudal surface (associated with the passage of tendons of the pes flexors in Aves; hypotarsus):

absent (0); developed as caudal projection with flat caudal surface (1); projection, with distinct crests and grooves (2); at least one groove enclosed by bone caudally (3). (ORDERED)

230. Plantar surface of tarsometatarsus excavated: absent (0); present (1).

231. Tarsometatarsal distal vascular foramen completely enclosed by metatarsals III and IV: absent (0); present (1).

232. Metatarsal I: straight (0); J-shaped, the articulation of the hallux is located on the same plane as the attachment surface of the metatarsal I (1); J-shaped; the articulation of the hallux is perpendicular to the attachment surface (2); the distal half of the metatarsal I is laterally deflected so that the laterodistal surface is concave (3).

233. Metatarsal II tubercle (associated with the insertion of the tendon of the m. tibialis cranialis in Aves): absent (0); present, on approximately the center of the proximodorsal surface of metatarsal II (1); present, developed on lateral surface of metatarsal II, at contact with metatarsal III or on lateral edge of metatarsal III (2). (ORDERED)

234. Metatarsal II, distal plantar surface, fossa for metatarsal I (fossa metatarsi I; Baumel and Witmer, 1993): absent (0); shallow notch (1); conspicuous ovoid fossa (2). (ORDERED)

235. Relative position of metatarsal trochleae: trochlea III more distal than trochleae II and IV (0); trochlea III at same level as trochlea IV, both more distal than trochlea II (1); trochlea III at same level as trochleae II and IV (2); distal extent of trochlea III intermediate to trochlea IV and II where trochlea IV projects furthest distally (3).

236. Metatarsal II, distal extent of metatarsal II relative to metatarsal IV: approximately equal in distal extent (0); metatarsal II shorter than metatarsal IV but reaching distally farther than base of metatarsal IV trochlea (1); metatarsal II shorter than metatarsal IV, reaching distally only as far as base of metatarsal IV trochlea (2).

237. Distal tarsometatarsus, trochlea in distal view: aligned in a single plane (0); metatarsal II slightly displaced plantarly with respect to III and IV (1); metatarsal II strongly displaced plantarly in respect to III and IV, such that there is little or no overlap in medial view (2).

238. Trochlea of metatarsal II broader than the trochlea of metatarsal III: absent (0); present (1).

239. Metatarsal III, trochlea in plantar view, proximal extent of lateral and medial edges of trochlea: trochlear edges approximately equal in proximal extent (0); medial edge extends farther (1).

240. Distal end of metatarsal II strongly curved medially: absent (0); present (1).

241. Digit IV phalanges in distal view, medial trochlear rim enlarged with respect to lateral trochlear rim: absent (0); present (1); greatly enlarged with the lateral trochlea reduced to a rounded peg (2).

242. Completely reversed hallux (arch of ungual phalanx of digit I opposing the arch of the unguals of digits II-IV): absent (0); present (1).

243. Size of claw of hallux relative to other pedal claws: shorter, weaker, and smaller (0); similar in size (1); longer, more robust, and larger (2).
244. Alula: absent (0); present (1).
245. Fan-shaped feathered tail composed of more than two elongate rectrices: absent (0); present (1).
246. Sternum, outermost trabecula: mainly parallel to the long axis of the sternum (0); clearly directed laterally (1).
247. Distal end of furcula relative to sternal margin of coracoid: proximal to or level with the sternal margin of the coracoid (0); well beyond the sternal end of the coracoid (1). When coracoid and furcula are not remained in natural position, then their proximodistal lengths are compared.
248. Scapula and coracoid: fused (0); unfused (1).
249. Scapula, acromion process length relative to the length of the humeral articular facet: less than half (0); nearly equivalent (1); longer but less than two times (2); more than two times longer (3); (ORDERED)
250. Alular digit (I), phalanx 1: longer than the phalanx 1 of digit II (0); shorter than or equivalent to the phalanx 1 of digit II (1).
251. Coracoid, width of the sternal end relative to the length along the shaft: approximately half or greater (0); between half to 1/3 (1); less than 1/3 (2).
252. Coracoid, sternal margin: convex (0); nearly straight (1); concave (2);
253. Humerus, deltopectoral crest, distal end recedes abruptly with the humeral shaft: present (0); absent (1)
254. Tibia/tarsal-formed condyles, intercondylar groove: mediolaterally broad, approximately 1/3 with of anterior surface (0); less than 1/3 width of anterior surface (1).
255. Metatarsal IV, distal extension of the metatarsal IV relative to the metatarsal III: shorter and proximal to the proximal margin of the trochleae III (0); shorter but reaching distally further than the proximal margin of the trochleae III (1); approximately equal or surpassing the trochleae III (2)
256. Reduced claw in digit IV: absent (0); present (1).
257. The ratio (tibiotarsus length/tarsometatarsus length): 2 or larger (0); between 2 and 1.6 (1); smaller than 1.6 (2). When distal tarsals are not fused with metatarsals, metatarsal III length is used.
258. Pedal digit, Penultimate phalanx, longer than preceding phalanges in each digit: absent (0); present (1).
259. Proximal phalanx of hallux, the longest non-ungual phalanx: absent (0); present (1).
260. Phalanx in digit IV: not as follows (0), the second and the third phalanges reduced and significantly shorter than the fourth phalanx (1), as before but with the proximal phalanx reduced to be nearly equal in length with the second and third phalanx (2).
261. Ungual in digit III, length relative to the tarsometatarsus: less than 20% the length of tarsometatarsus (0); 20% – 40% (1); extremely elongated and measuring more than 40% the length of tarsometatarsus (2); When metatarsals are not fused

with distal tarsals, the length of metatarsal III is used. The length of ungual represents the linear distance between proximal end (position equivalent to flexor process) and tip of the sheath. If the sheath is not preserved or disarticulated with bony ungual, it should be scored as "?". (ORDERED)

262. Surangular: not as follows (0); dorsal margin concave and ventral margin convex (1); sigmoid (2).

263. Premaxilla, preorbital portion occupying 60% or more the skull length: absent (0); present (1).

264. Pedal digit II: not as follows (0); much robust than the other digits (1)

265. Alular digit (I): long, exceeding the distal end of the major metacarpal (0); subequal (1); short, not surpassing this metacarpal (2). (ORDERED)

266. Tarsometatarsus, length compared to femur length: 0.6 or fewer (0); 0.8-0.6 (1); 1-0.8 (2);1 or greater (3).

267. Quadratojugal, shape: without horizontal process posterior to ascending process (reversed "L" shape) (0); with horizontal posterior process (i.e., inverted 'T' or 'Y' shape) (1).

268. Jugal and quadratojugal, fusion: absent (0); present, the two bones are not distinguishable from one another (1). (ORDERED)

269. Caudal vertebrae, change in morphology of free caudals along the tail: present, with distinct transition point from shorter centra with long transverse processes proximally to longer centra with small or no transverse processes distally (0); absent, vertebrae homogeneous in shape, without transition point (1).

270. Caudal vertebrae, location of transition point along the tail: begins distal to the 10th caudal vertebra (0); between the 7th and 10th caudal vertebra (1); proximal to the 7th caudal vertebra (2) (ORDERED).

271. Coracoid and scapula, angle between bones at glenoid: greater than or equivalent to 90° (0); smaller than 90° (1).

272. Ischium, distal end: continuous with the proximal shaft, rendering the cranial. margin of the ischium straight or weakly convex (0); directed cranioventrally toward the pubis, rendering the cranial margin concave (1).

273. Ischium, caudal margin with a dorsal process: located proximal to or close to the midpoint of the caudal margin (0); distal to the midpoint of the caudal margin (1).

274. Sternum, cranial margin with a pair of craniolateral processes: absent (0); present (1).

275. Premaxilla corpus: dorsoventral height greater than or equal to craniocaudal length (0); dorsoventral height smaller than craniocaudal length (1).

276. Furcula, omal tip: blunt or expanded (0); tapered (1).

277 Alular metacarpal, cranial margin: expanded cranioproximally and craniodistally, and constricted just before flare of articulation with digit (0); broadly convex (1).

278. Alular digit, phalanx 1: straight (0); bowed (1).

279. Major digit, phalanx 2: straight (0); bowed (1).

280. Minor digit: with four phalanges (0); less than four phalanges (1).
