# Supplementary material for: Bayesian tip dating reveals heterogeneous morphological clocks in Mesozoic birds
Source: R Soc Open Sci. 2019 Jul 24;6(7):182062. doi: 10.1098/rsos.182062 (PMC6689603; doi:10.1098/rsos.182062)
Supplement: Supplemental Figures [file rsos182062supp2.pdf]

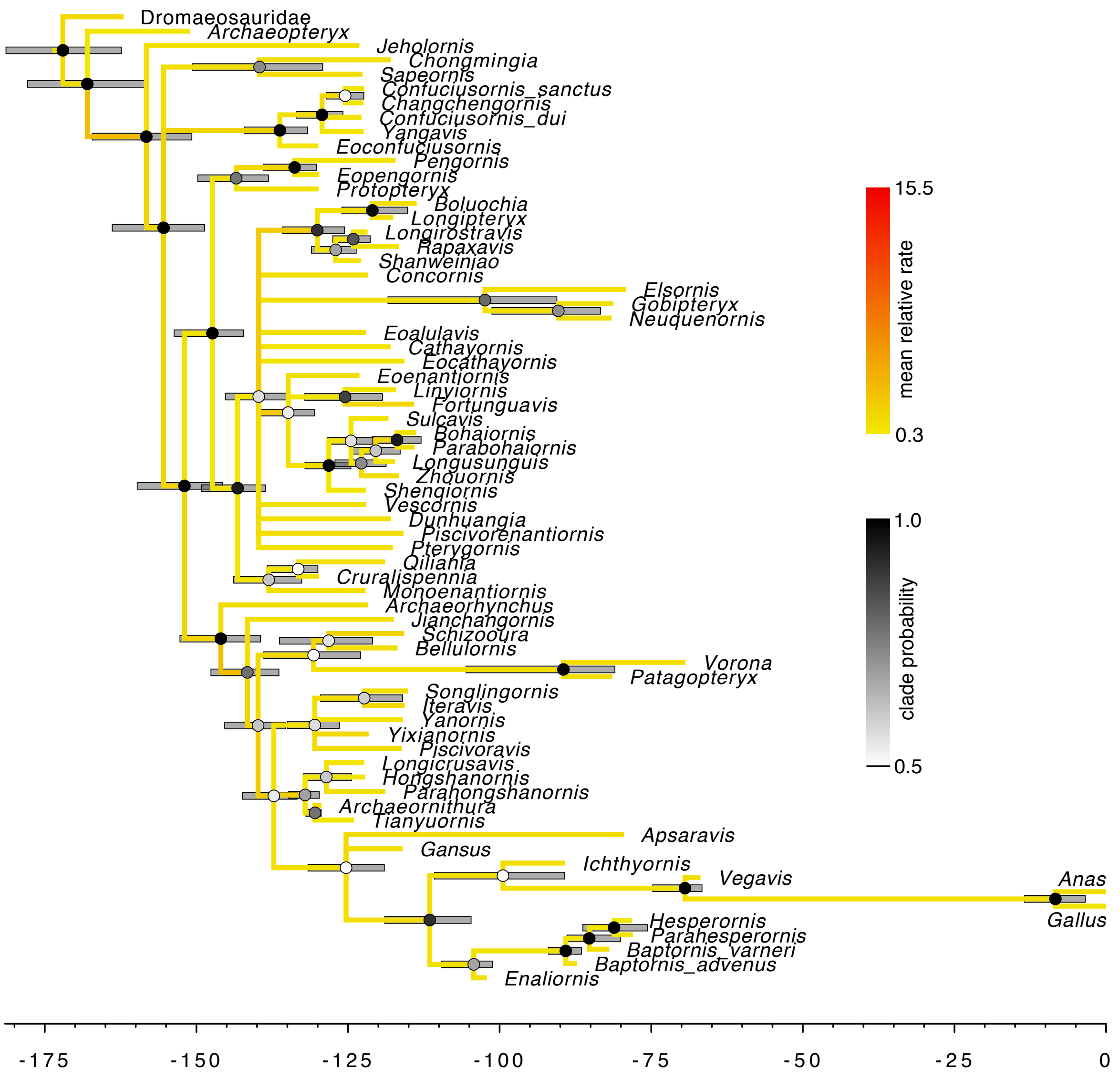

Figure S1. Dated phylogeny (time tree) of Mesozoic birds under the partitioned analysis. The color of the branch represents the mean relative clock rate of the **skull** at that branch. The node ages are the posterior medians and the error bars denote the 95% HPD intervals. The shade of the dot at an internal node represents the posterior probability of the corresponding clade. The following figures differ in the branch rates (heat color) while sharing the identical time tree.

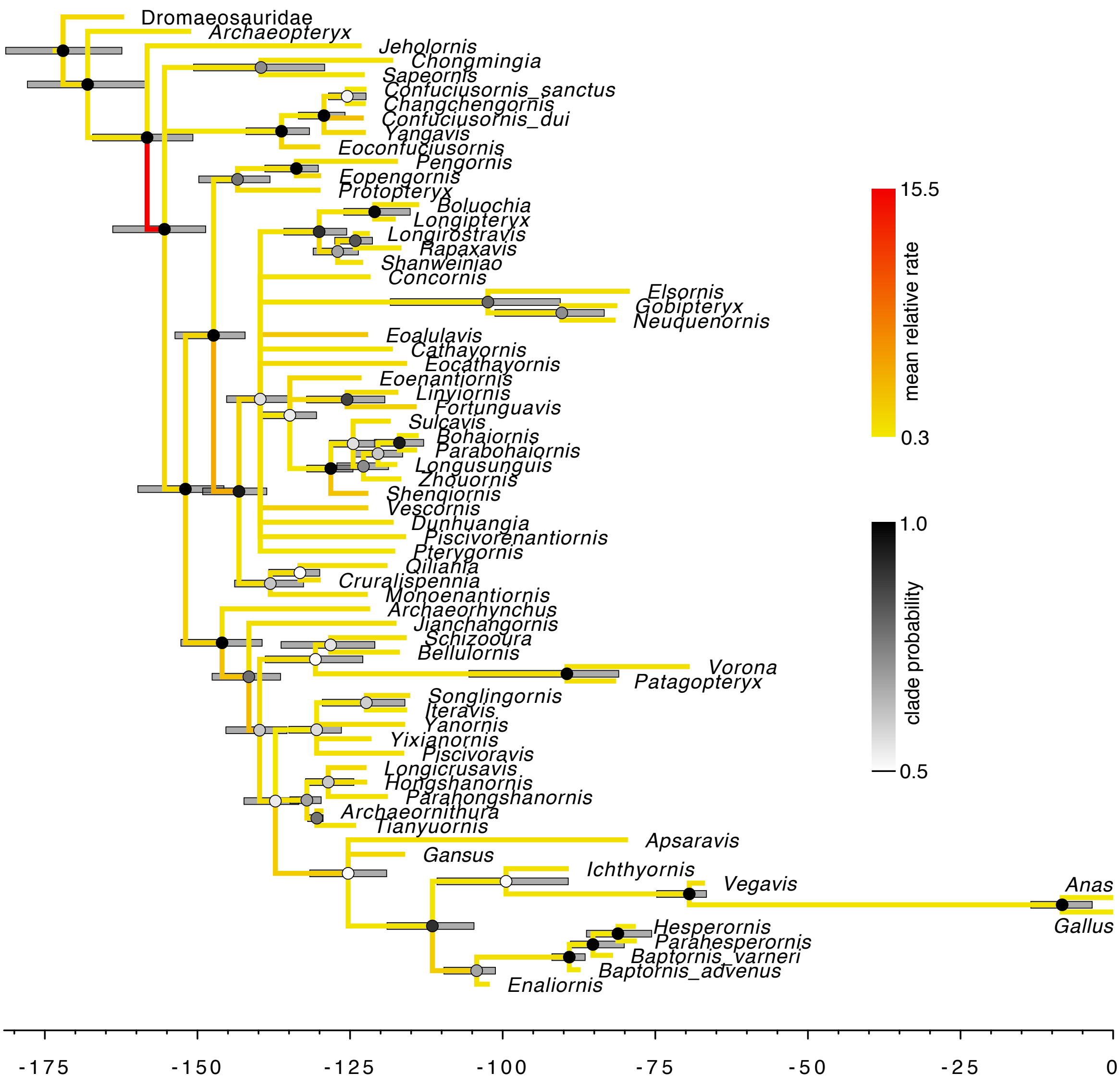

Figure S2. Dated phylogeny (time tree) of Mesozoic birds under the partitioned analysis. The color of the branch represents the mean relative clock rate of the **axial skeleton** at that branch. See legend to Figure S1.

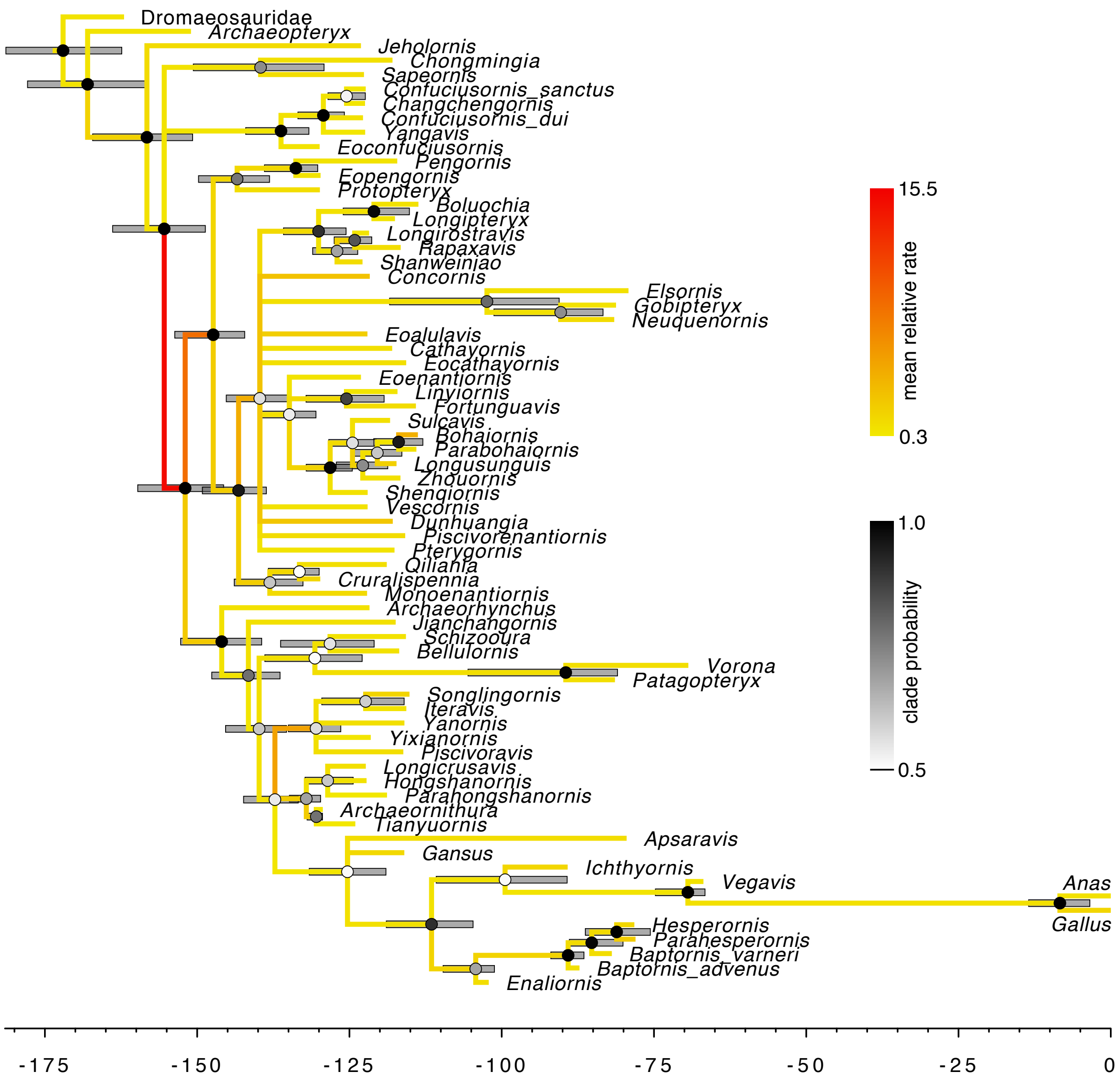

Figure S3. Dated phylogeny (time tree) of Mesozoic birds under the partitioned analysis. The color of the branch represents the mean relative clock rate of the **pectoral girdle and sternum** at that branch. See legend to Figure S1.

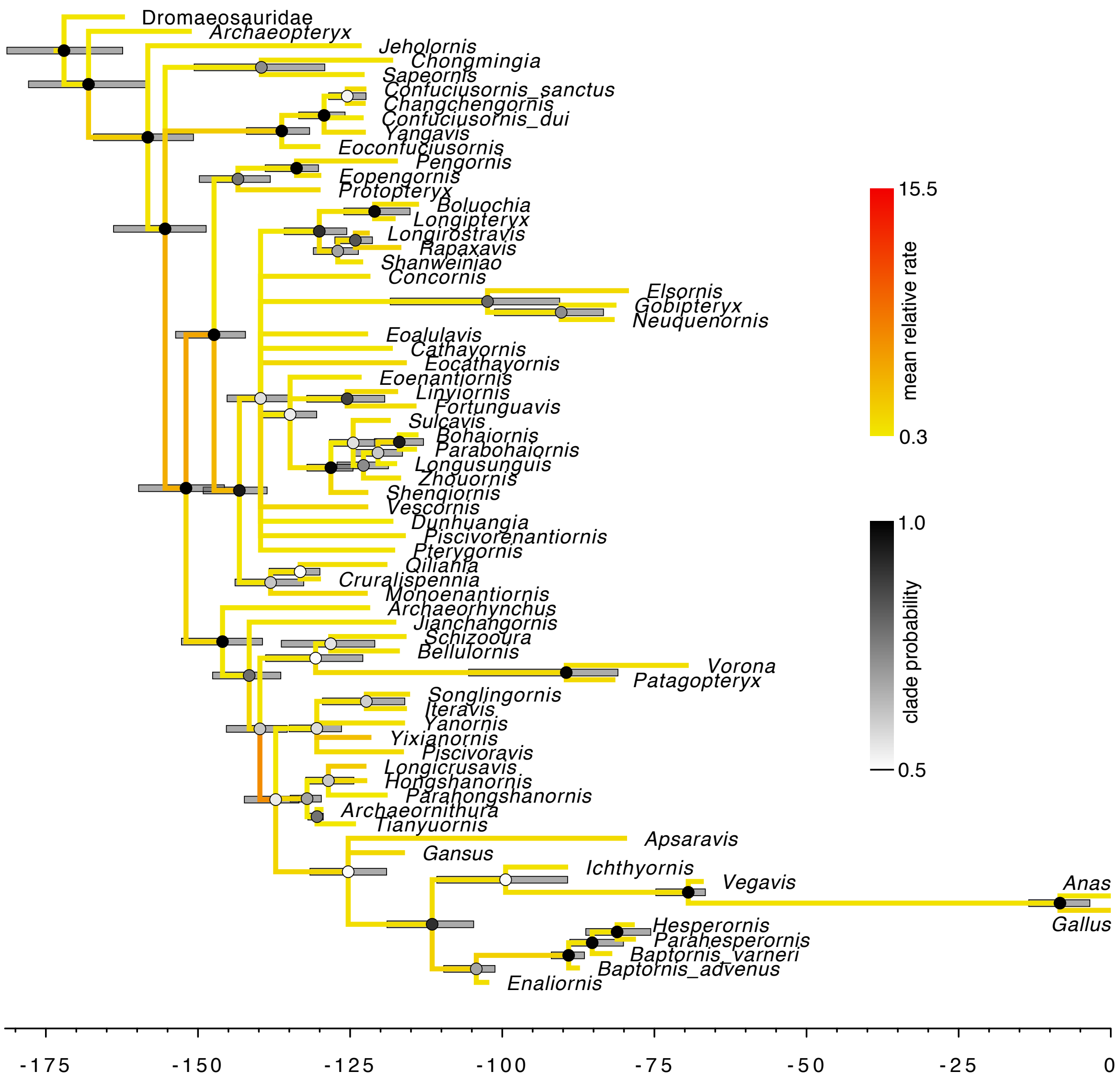

Figure S4. Dated phylogeny (time tree) of Mesozoic birds under the partitioned analysis. The color of the branch represents the mean relative clock rate of the **forelimb** at that branch. See legend to Figure S1.

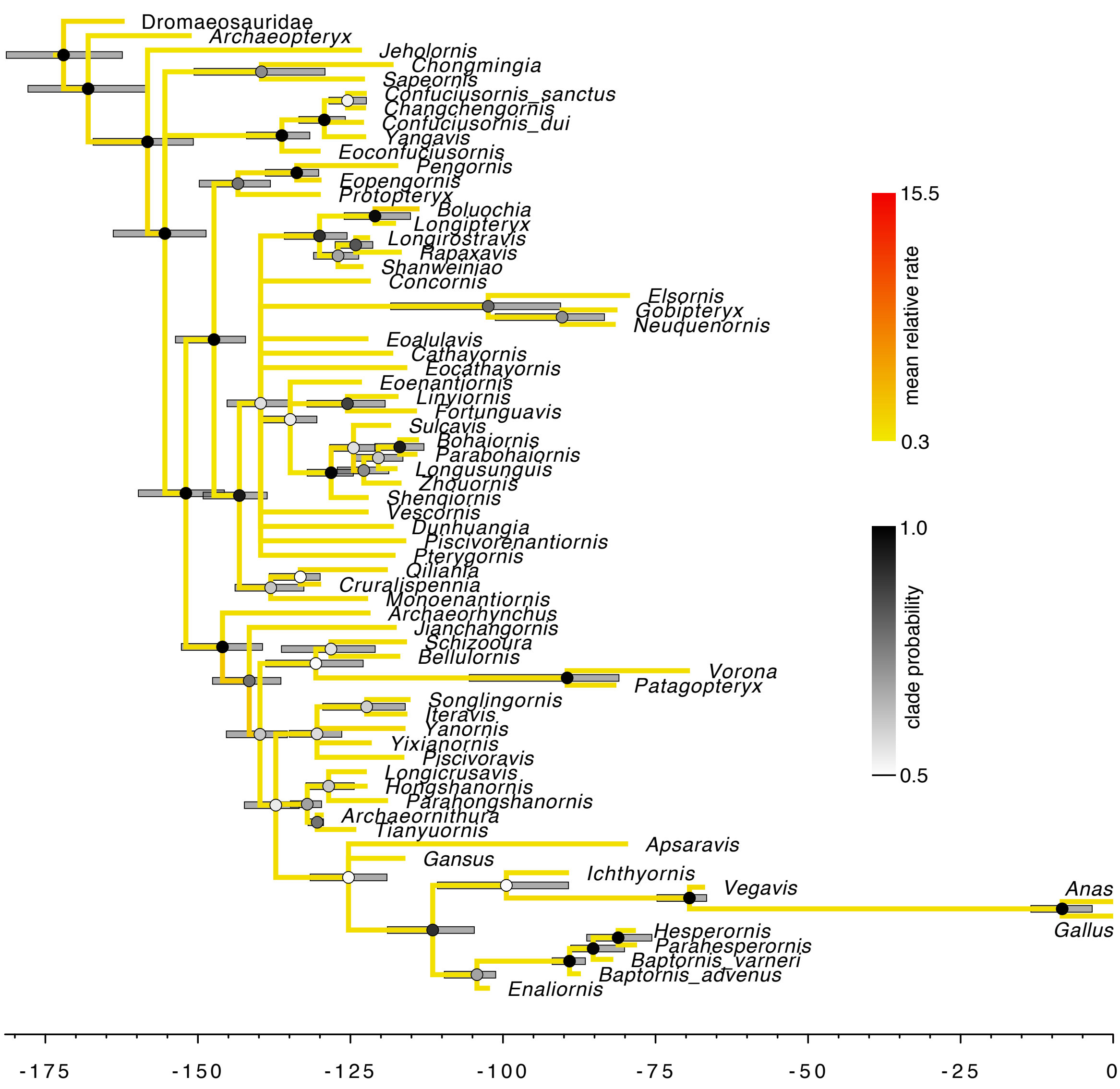

Figure S5. Dated phylogeny (time tree) of Mesozoic birds under the partitioned analysis. The color of the branch represents the mean relative clock rate of the **pelvic girdle** at that branch. See legend to Figure S1.

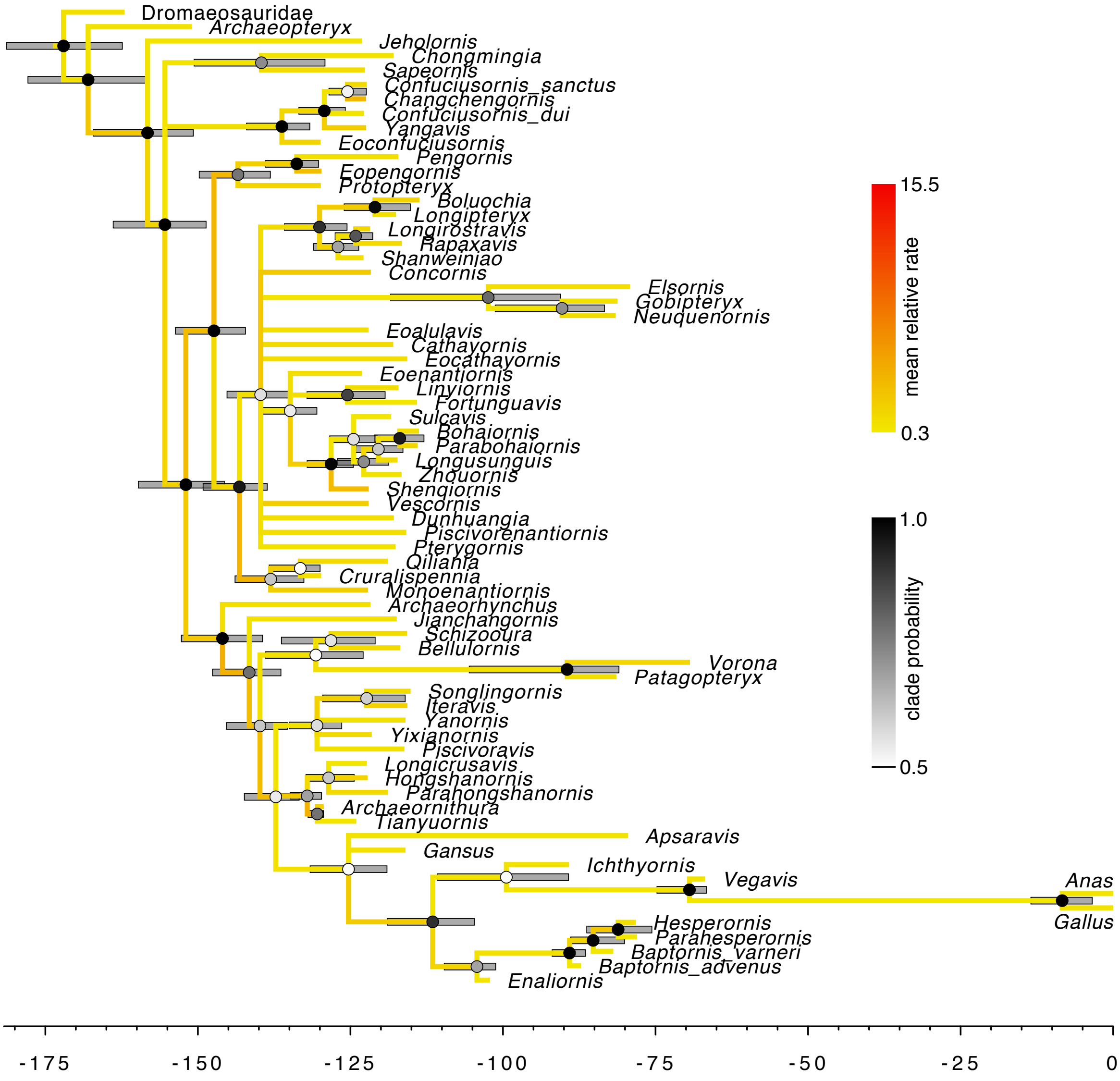

Figure S6. Dated phylogeny (time tree) of Mesozoic birds under the partitioned analysis. The color of the branch represents the mean relative clock rate of the **hindlimb** at that branch. See legend to Figure S1.
